# An integrated model of care to counter high incidence of HIV and sexually transmitted diseases in men who have sex with men – initial analysis of service utilizers in Zurich

**DOI:** 10.1186/1471-2458-8-180

**Published:** 2008-05-27

**Authors:** David LB Schwappach, Philip Bruggmann

**Affiliations:** 1 Research Institute for Public Health and Addiction, Zurich, Switzerland; 2 Faculty of Medicine, University Witten Herdecke, Germany; 3 ARUD Zurich, Association for Risk Reduction in the Use of Drugs, Zurich, Switzerland

## Abstract

**Background:**

As other countries, Switzerland experiences a high or even rising incidence of HIV and sexually transmitted infections (STI) among men who have sex with men (MSM). An outpatient clinic for gay men ("*Checkpoint*") was opened in 2006 in Zurich (Switzerland) in order to provide sexual health services. The clinic provides counselling, testing, medical treatment and follow-up at one location under an "open-door-policy" and with a high level of personal continuity. We describe first experiences with the new service and report the characteristics of the population that utilized it.

**Methods:**

During the 6-month evaluation period, individuals who requested counselling, testing or treatment were asked to participate in a survey at their first visit prior to the consultation. The instrument includes questions regarding personal data, reasons for presenting, sexual behaviour, and risk situations. Number and results of HIV/STI tests and treatments for STI were also recorded.

**Results:**

During the evaluation period, 632 consultations were conducted and 247 patients were seen by the physician. 406 HIV tests were performed (3.4% positive). 402 men completed the entry survey (64% of all consultations). The majority of respondents had 4 and more partners during the last 12 months and engaged in either receptive, insertive or both forms of anal intercourse. More than half of the responders used drugs or alcohol to get to know other men or in conjunction with sexual activity (42% infrequently, 10% frequently and 0.5% used drugs always). The main reasons for requesting testing were a prior risk situation (46.3%), followed by routine screening without a prior risk situation (24.1%) and clarification of HIV/STI status due to a new relationship (29.6%). A fifth of men that consulted the service had no history of prior tests for HIV or other STIs.

**Conclusion:**

Since its first months of activity, the service achieved high levels of recognition, acceptance and demand in the MSM community. Contrary to common concepts of "testing clinics", the *Checkpoint *service provides post-exposure prophylaxis, HIV and STI treatment, psychological support and counselling and general medical care. It thus follows a holistic approach to health in the MSM community with the particular aim to serve as a "door opener" between the established system of care and those men that have no access to, or for any reason hesitate to utilize traditional health care.

## Background

The incidence of HIV among men who have sex with men (MSM) in Switzerland increases since 2003 [1-3]. Whereas in 2003 159 HIV new infections were detected in MSM this number rose to 289 in 2006. Sexual contacts with other men accounts for about 40% of all HIV transmissions in Swiss men. The HIV prevalence in MSM younger than 30 years is 2%, in those aged over 30 years even 12% [4,5]. In sexually highly active MSM in Zurich every sixth is estimated to be HIV positive but only 2 out of 3 HIV positive MSM are aware of their serostatus [5]. As in other Western European countries, a significant increase in sexually transmitted infections (STI), e.g., syphilis, gonorrhoea and chlamydia infection, has been observed in Switzerland during the last years [6,7]. In addition to the burden associated with STIs, they also have a critical role in HIV seroconversion as the risk for transmission increases with the presence of mucosal lesions [8-11]. A not yet published on-site testing study in Zurich confirmed a clear rise in syphilis prevalence amongst gay men. Besides infectious diseases it is also the general health status of MSM that raises concerns. In a recent study conducted in the French part of Switzerland, gay men reported significantly more and particularly severe physical symptoms, short-term disability, mental disorders, and a higher prevalence of risk factors for chronic disease (high cholesterol, high blood pressure, high glucose, smoking), even after adjustment for differences in socio-demographic characteristics [12,13]. Elevated levels of tobacco smoking, alcohol consumption and use of illicit drugs amongst gay men have been reported in various studies throughout the world [14-20]. Finally, there is evidence that gay men are less likely to be satisfied with the health care they receive, in particular with respect to sexual health issues, and often do not disclose their sexual orientation to their providers [12,21-23]. However, a confidential and respectful relationship between providers and patients including open communication about sexual behaviour is essential in order to estimate STI and HIV risk and to provide adequate care to patients.

The serious health situation of MSM has led practitioners, researchers and decision-makers to discuss and develop alternative, low-threshold models of care for MSM at the community level. Such specialized health community clinics serving the lesbian, gay, bisexual and transgender population (LGBT) have a long tradition in the U.S. For example, the Fenway Community Health Centre was founded in 1971 [24]. There is, however, little experience with similar services in Europe. As Zurich has the largest gay community in Switzerland with a wide spectrum of gay specific venues, e.g., clubs and dark rooms, a large fraction of new HIV and STI infections amongst MSM occur there. With the aim to reduce rates of new HIV and STI infections and to provide comprehensive health services for MSM, an outpatient clinic for gay men was opened in June 2006: *Checkpoint Zurich*.

### Service description

The clinic is run jointly by the Zurich AIDS Federation and ARUD Zurich, a private organisation specialised in the care of drug addicts and experienced in treatment of their common health problems, such as infectious and psychiatric diseases. *Checkpoint Zurich *is very centrally located next to the main station, in walking distance from many bars, clubs, and dark rooms. Opening hours were initially on two evenings during the week (4 p.m. to 8 p.m.), but have been expanded to three evenings in 2007. Counselling and testing can be obtained anonymously. No appointments have to be fixed in advance and patients just can drop in for a consultation.

All tests and treatments are reimbursed by the Swiss basic health insurance but if clients prefer anonymous testing they have to pay on their own. Services provided to male sex workers are being paid for by the local government. The service was announced to MSM in gay bars and clubs, saunas, and gay media in Zurich. In addition, all registered general practitioners were sent an information leaflet.

Many existing HIV/STI testing centres across Europe are organised according to the VCT (Voluntary Counselling and Testing) principle. As they usually do not offer therapy or services unrelated to infectious diseases, referrals to other institutions or providers are often necessary. This transfer between two or even more institutions is time-consuming and requires patients to disclose intimate information repeatedly to new providers. Hence the risk of insufficient follow-up is high. In contrast, *Checkpoint Zurich *is an outpatient clinic managed by physicians. It provides the opportunity to obtain comprehensive care including counselling, testing, treatment and medical follow-up at one location and with a high level of personal continuity. Male nurses with a profound knowledge of and an accepting attitude towards gay sexual practices provide professional counselling. Physicians working at *Checkpoint Zurich *can draw on experiences gathered in the treatment of infectious diseases in drug addicts. Based on a patient's history and symptoms, clinical examinations, diagnosis of STIs, HIV tests (combination or rapid), and hepatitis A and B vaccinations are provided. Men with a history of sexual risk situation are offered tests for HIV and syphilis. Checkpoint offers tests for all other STIs, according to the patient's symptoms and history. Diagnostic procedures for Chlamydia and Gonorrhoea testing are based on the patient's presentation and symptoms. Patients with a recent risk of HIV infection are evaluated for post-exposure prophylaxis (PEP).

As usual in Switzerland, notifying partners is primarily seen as the responsibility of the newly diagnosed HIV/STI positive patient. However, partner notification is recommended to positive men during counselling and they are offered psychological support with this step.

In this study, we report first experiences with the new clinic and describe the clinic's performance and the characteristics of the population that utilized it. We provide details and outcomes of the services that were provided as well as clients' self-reported sexual behaviour, previous risk behaviour and rationales for seeking testing.

## Methods

The clinic started its activities in June 2006. Individuals that presented for counselling, testing or treatment were asked to complete a survey at their first visit. The survey was administered prior to the consultation while waiting to be called in. Clients were asked to complete the anonymous survey as a basis for their consultation and provided informed consent. Counsellors used survey responses as a starting point for discussing the client's situation and needs, but the written survey sheet used for data analysis was completely anonymous. Re-attending clients with repeat visits within the same episode, e.g., due to treatment, were identified using a code number and completed the survey only once at their initial visit. Re-attending clients with different episodes during the observation period – if any – could not be identified but were treated as individual cases in case they completed the survey more than once. Ethical approval was not necessary for this study. The questionnaire instrument includes questions regarding personal data, reasons for presenting, sexual behaviour, and risk situations. Descriptive statistics were used to analyze the main characteristics of the sample and to examine associations between selected variables. Comparisons were made using chi-square tests. A p-value of less than 0.05 was considered significant. All analyses were performed using STATA 10 software [25].

## Results

### Overall performance

During the evaluation period in the first 6 months of *Checkpoint Zurich *(12.6.2006–31.12.2006), the outpatient clinic was open for a total of 228 hours. In this period, 632 consultations took place and 247 patients were seen at least once by the physician. Counselling without testing was offered in 369 consultations. The vast majority of clients dropped in for a test, check or counselling and only very few consultations were fixed in advance. In the evaluation period, 241 HIV rapid tests and 165 HIV regular tests were performed. All positive rapid tests were confirmed with a regular p24AG/Ab test and a Western Blot. 14 HIV test were confirmed positive (3.4% of all tests). No false positive rapid test occurred. 184 tests for syphilis were performed with 7 positive results (3.8%) that were referred to treatment. 41 patients obtained therapy for Gonorrhoea or Chlamydia after being tested positive (234 tests, 18% positive). Patients with Gonorrhoea or Chlamydia showed different sites of positive testing. Urethral swabs were more often performed than rectal or pharyngeal ones. Alternatively to urethral swabs, Chlamydia and Gonorrhoea were also tested by urinary PCR. 36 Hepatitis vaccinations and 14 post-exposure prophylaxes (PEP) against HIV were provided.

### Survey results

During the evaluation period, 402 men completed the entry survey (64% of all *consultations*). The main reasons for non-response were lack of proficiency in the German language, repeated visits within the same episode, and, to a lesser extent, response refusal. The mean age of responders was 33 years (range 16–72 years). Nearly a quarter of men (22%) were of other than Swiss nationality. Their average length of stay in Switzerland was 15 years. Table 1 reports details of patients' characteristics. The majority of men had a history of one or more past HIV tests and reported a negative HIV serostatus as the last test result. Among those that had been tested for HIV previously, the last test was performed on average 3.3 years ago. The majority of men also reported prior tests for STI. The mean number of past tests for STI was 2.6. Among those that reported a history of STI (n = 115), urethral gonorrhoea (47%), condyloma (24%), Chlamydia infection (21%) and syphilis (16%) were most commonly specified. Subjects also reported their sexual behaviours (table 2). The majority of respondents had 4 and more partners during the last 12 months, engaged in either receptive, insertive or both forms of anal intercourse. Nearly half of the sample reported having unprotected anal intercourse at least infrequently. Having unprotected anal intercourse "always" or "frequently" was reported by 65 clients with respect to steady partners (16%), casual partners (5%) and unknown partners (3%).

**Table 1:** Self-reported details of responders' characteristics

Variable	n (%) responders*
Age	
Under 21 years	30 (7.5)
21 – 30 years	144 (35.8)
31 – 45 years	179 (44.5)
46 – 60 years	31 (7.8)
Older 61 years	11 (2.7)
Nationality	
Swiss	310 (77.1)
Other Europe	63 (15.7)
Asia	7 (1.7)
Central/South America	14 (3.5)
Other	3 (0.7)
Sexual orientation	
Homosexual	241 (60.0)
Heterosexual	106 (26.4)
Bisexual	54 (13.4)
Sexual contacts with ...	
Men only	131 (32.6)
Women only	36 (9.0)
Both men and women	37 (9.2)
Relationship	
Living in a monogamous relationship	124 (30.8)
Living in an open relationship with arrangements	78 (19.4)
Living in an open relationship without arrangements	19 (4.7)
Living alone	150 (37.3)
Result of last HIV test	
Negative serostatus	309 (76.9)
Positive serostatus	2 (0.5)
Never been tested	78 (19.4)
Hepatitis A vaccination status	
Not vaccinated	92 (22.9)
Vaccinated	183 (45.5)
Vaccination status unknown	112 (27.9)
Hepatitis B vaccination status	
Not vaccinated	84 (20.9)
Vaccinated	199 (49.5)
Vaccination status unknown	102 (25.4)
Number of past tests for sexually transmitted infections (STI)	
0	89 (22.1)
1–2	138 (34.3)
3 and more	141 (35.1)
History of sexually transmitted infections (STI)	
No	277 (68.9)
Yes	115 (28.6)

*n varies per item due to item-non-response. % = relative to the entire sample (n = 402)

**Table 2:** Self-reported sexual behaviours

Variable	n (%) responders*
Practiced sexual techniques, last 12 months	
Anal intercourse	
insertive only	69 (17.2)
receptive only	32 (8.0)
both	168 (41.8)
Frequency of unprotected anal sex	
never	227 (56.5)
seldom	118 (29.4)
frequently	23 (5.7)
always	8 (2.0)
Vaginal intercourse	132 (32.8)
Oral intercourse	
insertive oral sex only	42 (10.4)
receptive oral sex only	24 (6.0)
both	260 (64.7)
Oro-anal sex	
insertive only	9 (2.2)
receptive only	21 (5.2)
both	100 (24.9)
Fisting	
Insertive only	21 (5.2)
receptive only	7 (1.7)
both	10 (2.5)
Sadomasochistic practices	18 (4.5)
Number of sex partners, last 12 months	
0	21 (5.2)
1	47 (11.7)
2–3	115 (28.6)
4–10	133 (33.1)
> 10	86 (21.4)
Number of sex partners for anal intercourse, last 12 months	
0	132 (32.8)
1	82 (20.4)
2–3	94 (23.4)
4–10	62 (15.4)
>10	32 (8.0)
Number of sex partners for unprotected anal intercourse, last 12 months	
0	258 (64.2)
1	91 (22.6)
2–3	45 (11.2)
4–10	8 (2.0)
>10	--

*n varies per item due to item-non-response. % = relative to the entire sample (n = 402)

When asked for the frequency of sexual activity, 3% reported to have daily sexual contacts, several contacts per week (28%), one contact per week (25%) and 35% reported less than weekly sexual activity with a partner. When asked for satisfaction with their sexual life, men scored on average 6.8 points measured on a 10-point Likert-scale with 1 indicating "not all satisfied" and 10 indicating "very satisfied". Many men reported to meet new partners for sexual contacts on the Internet (39%); 154 met new partners at a bar or club (38%); 89 through friends (22.1%); 85 in foreign countries or during travel (21%); 84 in a sauna (21%); 51 at sexclubs (13%); 31 at porn cinemas, parks or public toilets (8%); and 4% met new partners in various other locations. More than half of the responders used drugs or alcohol to get to know other men or in conjunction with sexual activity (42% infrequently, 10% frequently and 0.5% used drugs always). The most common substances used in conjunction with sex were alcohol (41%), followed by cannabis (13%), cocaine (5%), methylenedioxymethamphetamine (ecstasy; 5%), sildenafil (viagra; 4%) and other substances (5%), mainly inhalant nitrites (poppers), crystal methamphetamine (crystal) and Gamma-Hydroxybutyrate (GHB/GBL).

The main reasons for requesting HIV or STI testing were a prior risk situation (46.3%), followed by routine screening without a prior risk situation (24.1%) and clarification of HIV/STI status due to the beginning of a new relationship (29.6%). 2.5% of clients reported a prior STI as rationale for requesting HIV testing while 2 men (0.5%) were diagnosed with HIV previously and asked for STI testing. Details on the reported previous risk behaviours are presented in table 3. The most common risk behaviours reported by respondents were oral uptake of body fluids, unprotected insertive anal intercourse without ejaculation and unprotected vaginal intercourse with ejaculation. Reported risk situations most commonly occurred longer than 15 days ago and in private locations with anonymous partners of unknown HIV serostatus. On average, men estimated their risk for HIV or STI infection as 3, measured on 10-point Likert scale (0 = Very low; 10 = Very high). 29% of men reported one or more symptoms that may be associated with primary HIV infection (oral lesions or infection 11%; night sweat 10%; diarrhoea 9%; fever 8%; muscle and joint pain 8%; vomiting 2%;). As can been seen from table 1, a fifth of men that consulted the service had no history of prior tests for HIV or other STIs. To learn more about differences between groups of utilizers, two distinct subgroups were created: those with a history of both, prior tests for HIV and STI and those without a history of either test (table 4). Compared to men in the 'no test group', men in the 'test group' were significantly older, were more likely to self-identify as gay, and were less likely to live in a stable relationship. However, there were no differences between the groups in terms of the motivation for consultation or characteristics of prior risk situations.

**Table 3:** Details of self-reported previous risk behaviour as rationale for seeking testing

Variable	n (%) responders*
*Risk behaviour (multiple responses allowed), referenced to time and location as below*	
Unprotected anal intercourse	
Without ejaculation	
insertive	48 (11.9)
receptive	26 (6.5)
With ejaculation	
insertive	35 (8.7)
receptive	23 (5.7)
Condom failure	
insertive	21 (5.2)
receptive	14 (3.5)
Unprotected vaginal intercourse	
Without ejaculation	30 (7.5)
With ejaculation	43 (10.7)
Condom failure	19 (4.7)
Oral uptake of body fluids (blood, sperm)	75 (18.7)
Other risk behaviour	39 (9.7)
*Risk situations*	
Partners involved in the risk situation (multiple responses allowed)	
Steady partner	79 (19.7)
Casual partner	100 (24.9)
Anonymous partner	148 (36.8)
HIV serostatus of involved partners	
Positive serostatus	28 (7.0)
Negative serostatus	107 (26.6)
Serostatus unknown	190 (47.3)
Location of risk situation	
Privat location (at home)	172 (42.8)
Bathhouse/Darkroom	52 (12.9)
Park/cruising area	19 (4.7)
Private party	10 (2.5)
Other location	42 (10.4)
Time of risk situation	
0 – 3 days ago	21 (5.2)
4 – 14 days ago	30 (7.5)
15 days – 3 months ago	109 (27.1)
3 – 6 months ago	93 (23.1)
> 6 months ago	52 (12.9)

*n varies per item due to item-non-response. % = relative to the entire sample (n = 402)

**Table 4:** Differences between prior and non-prior testors

Variable	n (%) responders		*p*
	Testors* (n = 276)	Non-testors (n = 65)	

Mean age, years	34	28	<0.001
Sexual orientation: Self-defined as 'gay'	68%	35%	<0.001
Living in a stable relationship	30%	46%	0.021
Swiss nationality	79%	77%	0.729
Having had more than 10 sexual partners in the preceding 12 months	27%	19%	0.239
Prior risk situation as motivation to seek testing	45%	49%	0.566
New relationship as motivation to seek testing	29%	40%	0.084
Routine check-up as motivation to seek testing	26%	17%	0.121
Risk situation with anonymous partner	42%	48%	0.687
Use of drugs in conjunction with sexual activity	54%	48%	0.448
Risk situation longer than 3 months ago	45%	48%	0.688
Estimated risk for HIV or STI infection (10-point Likert scale (0 = Very low; 10 = Very high))	3.1	2.5	0.043

* Testors were defined as men with a history of HIV and any other STI. Non-testors had no history of either test.

## Discussion

As other countries Switzerland experiences a high or even rising incidence of HIV and sexually transmitted diseases amongst MSM. The *Zurich Checkpoint *was established to offer men a comprehensive prevention, testing, counselling, and treatment facility provided by gay health care professionals based on an "open-door" policy. Since its first months of activity, the service achieved high levels of recognition, acceptance and demand in the MSM community and now plays an important role in HIV/STI counselling and detection. In 2007, 101 new HIV infections in MSM were recorded in Zurich (303 in entire Switzerland). Of these 101 new infections, 57 (56%) were detected at *Checkpoint Zurich*. Another 14 men (14%) newly diagnosed with HIV at other sites in Zurich (e.g., laboratories, general practitioners, testing facilities at the university hospitals) were referred to *Checkpoint *after the initial diagnosis for further counselling or treatment. In other words, after 6 months of activity Checkpoint was involved in the care of 70% of MSM in Zurich newly diagnosed with HIV. As reported, demand was high and so the initial person-hours (counsellors) were expanded from 640 in 2006 to 1570 (1100 stationary and 470 mobile testing and counselling hours) in 2007 and 1800 (1200 stationary and 600 mobile) in 2008. Person-hours of physicians increased from 228 in 2006 to 453 in 2007 and 462 in 2008. Acceptance within the community was also confirmed in a recent survey study among 379 gay men in Zurich, not necessarily clients of the *Checkpoint*. In a discrete choice experiment involving vignettes with alternative service descriptions, responders strongly preferred smoking cessation services provided by *Checkpoint *compared to that offered by other providers, irrespective of programme content and characteristics of fellow participants [26,27].

While demand was generally high in 2006, only few HIV-positive men requested care at the new service, mainly for STI testing. This is not surprising since HIV-positive men were not actively targeted in 2006 due to limited opening hours. However, this situation has changed since 2007 as with expanded opening hours Checkpoint is now also capable to offer long term HIV follow up, monitoring and treatment.

Around 3.4% of HIV tests were positive, a relatively high number in comparison with testing centres at the university hospitals. In 2006, 4012 anonymous tests for HIV were conducted at the University hospital Zurich with 15 positive results (0.4%, personal communication). However, with respect to the estimated HIV prevalence of 15% in the MSM population with sexual activity with anonymous partners the number of positive tests in the first half-year period of *Checkpoint *seems still quite low. In an on-site cross-sectional study among gay/bisexual men conducted at 24 venues in Zurich in 1998, self-reported HIV status was recorded and compared to the results of HIV tests in saliva. 11.6% of participants were tested HIV-positive [5]. However, amongst men with a positive test result the majority were aware of their positive HIV serostatus (69% of those with a positive test, 8% of all participants). 3.6% of men that were tested positive were unaware of their HIV status. This figure is very close to the prevalence among men seeking testing reported in our study but the prevalence among subgroups of men may be much higher. For example, only few men in our sample reported "private parties" as site of the risk situation that motivated them to seek testing. In contrast, counsellors and outreach workers observe that "barebacking parties" with and without serosorting rules are becoming increasingly popular among gay men in Zurich. It seems that men engaging in this highly risky behaviour were not yet fully reached by the new service in 2006. New outreach strategies with on-site testing at sex parties, in dark rooms, saunas and cruising areas like motorway rest stops were started in 2007 under the name of *Checkpoint mobile*. Preliminary results indicate that the number of HIV tests performed increased by 50% with mobile testing while the prevalence of positive results nearly doubled. In the sample of 2006, many men had a history of at least one STI test in the past and one third had suffered any STI previously. This underlines the considerable burden that STI other than HIV pose to the health of MSM. In the perception of counsellors there is also increasing awareness towards STI and the fraction of subjects who seek testing and treatment explicitly due to concerns over STI other than HIV is steadily rising.

This study also has a number of limitations and results should therefore interpreted with care: The most important limitation is that the study is purely descriptive and we cannot present data on a control group, e.g., MSM that demand care elsewhere in Zurich. As the survey was mainly used for counselling purposes and was therefore administered prior to consultations and treatments, it was not possible to include questions relating to patients' evaluation of the care they received, e.g., in terms of satisfaction with the new service. We therefore needed to rely on demand as a marker of acceptance within the MSM community. Another limitation relates to the response rate. Since we lack data of non-responders, we cannot draw conclusions regarding potential differences between responders and non-responders. However, it is worth noting that the response rate of 64% relates to responses/consultations and thus underestimates the true response rate due to repeat visits in the same episode. The main reasons for true non-response were language barriers and low literacy, in particular in migrants from non-European countries, e.g., South America. To overcome this deficit, survey versions in a number of languages have already been developed and are now being administered computer-based with the option to get support from staff. Finally, we were not able to identify survey responses of clients re-attending with new episodes, if any. While this fraction is considered to be negligible given the short observational period, a client tracking system for matching survey responses of the same individual will be implemented in future evaluations.

*Checkpoint Zurich *as a model of comprehensive care for MSM has already been reproduced in other European cities in 2007, e.g., in Munich. *Checkpoint Munich *is run by the Munich AIDS foundation and offers similar services. Other metropolitan areas, in particular in Germany, are currently adopting the Checkpoint concept to their needs (cities of Cologne, Dresden) or have already reproduced parts of it. For example, the concept of *Checkpoint mobile *has already been transferred to Berlin. The advantage of this model in comparison to traditional VCT projects is the opportunity to offer diagnosis, therapy and follow-up at the same location through the same team with a considerably lower rate of positive tested patients that do not obtain treatment.

Our data also provide further evidence that substance use, in particular alcohol consumption, is common amongst MSM. It represents a major threat to MSM health and is likely to play an important role in HIV and STI risk-taking behaviour. These data are confirmed by other national and international studies [16,28,29]. For example, Wang et al. report from a survey study amongst gay men living in the French part of Switzerland that one out of ten gay men reported consuming high daily quantities of alcohol (≥ 60 g/day) in the past 4 weeks and gay men had a more than(threefold adjusted odds ratio of any drug use within the past 12 months compared to the general male population [12]. Nearly half of the respondents fulfilled the criteria for at least one of five DSM-IV disorders (12 month prevalence: major depression (19.2%), specific phobia (12.6%), social phobia (13.5%), alcohol dependence (11.4%), drug dependence (7.3%)) [13]. These data are deeply worrying. Hence, concepts for psychosocial screening and counselling are currently being developed to counteract substance abuse in the MSM community.

## Conclusion

In contrast to common concepts of "testing clinics", the *Checkpoint *service also provides post-exposure prophylaxis, HIV and STI treatment, psychological support and counselling and general medical care. It thus follows a holistic approach to health in the MSM community with the particular aim to serve as a "door opener" between the established system of care and those men that have no access to, or for any reason hesitate to utilize, traditional health care. Mobile testing services and interventions for issues other than HIV and STI are consequently expanded to reach high-risk populations and target other health concerns and high prevalence of mental disorders amongst MSM.

## Competing interests

The authors declare that they have no competing interests.

## Authors' contributions

DLBS and PB conceived of the study and participated in its design. PB coordinated the study group. DLBS performed the statistical analysis. Both authors drafted the manuscript, and read and approved the final manuscript.

## Pre-publication history

The pre-publication history for this paper can be accessed here:


